# Medium-sized posterior fragments in AO Weber-B fractures, does open reduction and fixation improve outcome? the POSTFIX-trial protocol, a multicenter randomized clinical trial

**DOI:** 10.1186/s12891-017-1445-0

**Published:** 2017-02-23

**Authors:** Sander Verhage, Peer van der Zwaal, Maarten Bronkhorst, Huub van der Meulen, Sanne Kleinveld, Sven Meylaerts, Steven Rhemrev, Pieta Krijnen, Inger Schipper, Jochem Hoogendoorn

**Affiliations:** 1 Trauma-unit Haaglanden MC, The Hague, The Netherlands; 2 Department of Traumasurgery, Haga Ziekenhuis, The Hague, The Netherlands; 3 Department of Traumasurgery, Leiden University Medical Center, Leiden, The Netherlands

**Keywords:** Trimalleolar fracture, Posterior malleolar fracture, Posterior fragment fixation, Ankle fracture, Posterolateral approach

## Abstract

**Background:**

Guidelines for treatment of the posterior fracture fragment in trimalleolar fractures are scarce and show varying advices. Did the increasing size of the posterior fragment seem to relate to worse outcome in the past, nowadays this has changed to the amount of dislocation of the posterior fragment post-operatively. Despite many retrospective cohort studies and some prospective cohort studies, no consistent guideline could be derived from the current literature.

**Methods:**

The POSTFIX-study is designed as a multicenter randomized clinical trial to analyse the effects of anatomical reduction and fixation of the posterior fragment in AO 44-B3 fractures with medium-sized posterior fragment. A total of 84 patients will be included and online allocated to either anatomical reduction and fixation of the posterior fragment via the posterolateral approach (*n* = 42) or no fixation of the posterior fragment (*n* = 42). The concomitant fractured medial and lateral malleoli are treated according to the AO-principles. Functionality of the ankle as measured by the AAOS-questionnaire (American Association of Orthopaedic Surgeons) 1 year post-operatively was set as primary outcome. Main secondary outcome measures are the AAOS-questionnaire 5 years postoperatively and osteoarthritis as measured on plain radiographs 1 year and 5 years post-operatively. The Olerud and Molander score, the AOFAS-score, the VAS-pain, the Euroqol-5D and Range of Motion by physical examination will also be evaluated during the follow-up period.

**Discussion:**

The POSTFIX-trial is the first high quality multicenter randomized clinical trial worldwide to analyse the effects of anatomical fixation of the posterior fragment in trimalleolar fractures. New guidelines on anatomical reduction and fixation of the posterior fragment can in future be based on the results of this trial.

**Trial registration:**

This trial is registered on ClinicalTrials.gov with reference number: NCT02596529. Registered 3 November 2015, retrospectively registered.

## Background

The optimal treatment of ankle fractures with involvement of the posterior malleolus remains a subject of debate. Despite a large amount of literature on the role of the posterior malleolus in a so-called trimalleolar fracture, there are no clear guidelines for its treatment. Its size was taught to be the leading indicator for the need of fixation of the fragment [1, 2]. Most orthopaedic surgeons consider a posterior malleolar fracture fragment larger than 25 to 33% of the joint surface an indication for fixation. Interestingly, after careful evaluation of the available literature [3–8], there does not seem to be solid substantiation of this assumption.

Theories about the relation between the size of the posterior fracture fragment and outcome are partially based on biomechanical studies. First, a large posterior fragment was thought to lead to posterior instability and therefore to worse outcome on the long term. Several cadaveric ankle studies however, showed different cutoff values ranging from 25 to 33% of the involved articular surface [9, 10]. Other studies could not prove the theory of posterior instability in cadaveric ankles [11–14]. Later cadaveric studies suggested a shift of contact pressure pattern in case of a posterior malleolar fracture and therefore the early induction of increased in post-traumatic osteoarthritis [15, 16].

Several retrospective cohort studies showed no clear relation between fragment size and functional outcome. Jaskulka et al. and Langenhuijsen et al. found a worse long-term outcome (follow-up 5.7 and 6.9 years) in fragments larger than 5 and 10% of the involved articular surface respectively (3,5]. Broos et al. found a worse outcome after 1 year in patients with posterior fragments larger than 33% of the involved articular surface [6]. De Vries et al. found no relation between size and functional outcome but he suggested a worse functional outcome in case of a posterior malleolar fracture dislocation [4].

More recently two large retrospective cohort studies on the influence of joint congruency in posterior malleolar fractures were published. The first was performed by Xu et al., who studied 102 trimalleolar fractures with a mean follow up of 2.8 years [8]. There was no relation between fragment size and radiological or functional outcome (AOFAS). However, they found a worse functional outcome if a persisting tibiotalar step-off was present after open reduction and internal fixation. Therefore they advised to anatomically restore the articular surface, especially when fragment size involves than 25% of the joint surface [8].

Drijfhout et al. performed a retrospective cohort study of 131 trimalleolar fractures with a mean follow-up of 7.3 years [7]. Functional outcome was worse in trimalleolar fractures with a medium-sized (5–25%) or large (>25%) posterior fragment compared to small fragments (<5%). A postoperatively persisting step-off ≥1 mm of medium-sized or large posterior fragments showed to be the most important predictor for the development of osteoarthritis [7]. Restoration of the tibiotalar articular surface therefore seems essential in medium-sized and large fragments.

As shown, no consistent advice is found in the literature as to which fragment size of the posterior malleolus should be internally fixed. Currently, according to AO guidelines [17] fixation of posterior fragments is indicated if there is a displaced fragment larger than 25% of the involved articular surface or if instability is persistent after reduction of the lateral injury [17]. Traditionally, reduction of these larger fragments is performed indirectly, followed by percutaneous screw fixation in anterior-posterior direction. It is often challenging to achieve an anatomical reduction and fixation of smaller fragments using the percutaneous method. Recently, direct exposure of the posterior tibia via a posterolateral approach, followed by open reduction and fixation with screws in posterior-anterior direction or an antiglide plate, is advocated by several authors [18–21]. This approach, with the patient in prone position, allows good visualization of the fracture, articular anatomical reduction, and solid fixation. Another advantage is that even small posterior fragments can be addressed. Several case series published favourable results; few major wound complications, good functional outcomes, and rarely a need for reoperation [22, 23].

To test the hypothesis that anatomical reduction and fixation of medium-sized posterior fragments via the posterolateral approach is meaningful, this multicenter randomized clinical trial was designed. As far as we know, this is the first randomized controlled trial on fixation of the posterior fragment in trimalleolar fractures worldwide.

## Methods/design

### Aim of the study

Anatomical reduction and fixation of medium-sized posterior malleolar fractures leads to a stable and anatomical fixation [22, 23]. In this study, we hope to prove that anatomical reduction and fixation of medium sized posterior fragments leads to less osteoarthritis and better functional outcome in patients having a trimalleolar fracture.

### Study design and setting

This study comprises a multicenter randomized controlled clinical trial in three teaching hospitals in the Netherlands, evaluating functional outcome using the AAOS foot and ankle questionnaire after 1 year in AO-44B3 trimalleolar fractures with a medium-sized (5–25%) posterior fragment. Patients will be randomized between; 1. Open reduction and fixation with additional fixation of the posterior malleolus via the posterolateral approach or 2. Open reduction and fixation without fixation of the posterior fragment. Two level-1 traumacenters (MCH Westeinde, Haga ziekenhuis) and one level-2 traumacenter (Bronovo hospital) participate, which are all centers in The Hague. The study will be open for inclusion from 2015 till 2019, the follow up will be completed in 2024. The Medical Ethics Committee South West Netherlands approved of the study protocols (protocol number 15-040). The study is registered at ClinicalTrials.gov (Number NCT02596529).

### Study population

All patients presenting at the emergency department with an AO-44B3 trimalleolar fracture with a medium-sized (5–25%) posterior fragment are asked to participate in the study. The initial decision on inclusion is based on review of pre-operative radiographs since this best resembles daily practice. The pre-operative X-rays are judged by 2 observers to confirm eligibility for the study. Fragment size is measured on plain lateral radiographs at tibiotalar joint level. A computed tomography scan is made in order to assess intra-articular fragments and correlation with pre-operative X-rays and posterior fragment size.

The inclusion criteria are: age between 18 and 75 years, trimalleolar AO-44B3 fracture with medium sized (5–25% of the distal tibial articular surface) posterior fragment and first ankle fracture of affected side. Excluded are multitraumatized patients (ISS > 16), patients with multiple fractures or open fractures, ankle fracture of same side in medical history, with pre-existent disability or mobility problems (need of walking gait), where follow-up takes place in another hospital and with insufficient understanding of the Dutch language. A detailed list of in- and exclusion criteria is presented in Table 1.

**Table 1:** Inclusion and exclusion criteria

Inclusion criteria	
1. >18 and <70 years at time of inclusion	
2. AO 44-B3 fracture with medium-sized posterior fragment	
3. First ankle fracture of affected side	
Exclusion criteria	
1. Severely traumatized patients (ISS > 16)	
2. Multiple fractures	
3. Ankle fractures of the same ankle in the history	
4. Patients with pre-existent mobility problems	
5. Pre-existent disability	
6. Patients with follow-up in another hospital	
7. Insufficient understanding of the Dutch language	

### Recruitment, informed consent and randomization

**Fig. 1:**
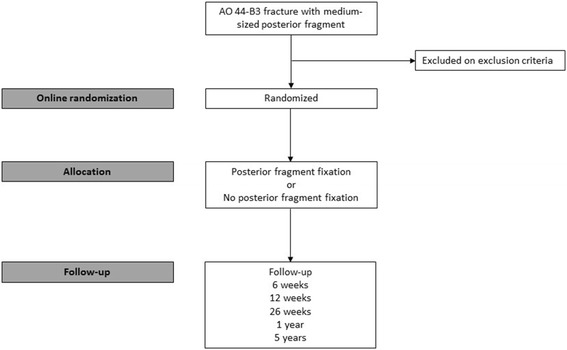
Flow-chart of inclusion and randomisation

Patients are first seen at the Emergency Department and will receive the study information from the attending surgeon or surgical resident. All patients will be informed about the potential risks and complications of both fix and non-fix management of the posterior malleolus. If the patient is eligible for inclusion (based on fragment size measured on plain lateral X-ray) and willing to participate, the research coordinator includes the patient within 1 week. All participants provide written informed consent. After inclusion, participants are allocated to one of the two randomisation groups by an online randomization program in blocks of 6 or 8 patients. A flow-chart of inclusion and randomization is shown in Fig. 1. Blinding is not possible because the two different soft tissue approaches in the treatment protocols indicate which type of surgical fixation is performed.

### Treatment protocol

All operative interventions are performed by experienced surgeons familiar with both treatment protocols and fixation techniques. Pre-operatively 1 gram Cefazoline® prophylaxis is administered. Dependent form surgeon’s preference, patients will be operated with a thigh tourniquet. In case of non-fixation of the posterior fragment, patients are operated in supine position. The lateral malleolus is fixed with two lag screws and/or lateral plate fixation. The medial malleolus is fixed with two cancellous screws or tension band wiring. In case of allocation to the fixation-group, the participant is operated in prone position. The posterolateral approach is used for fixation of both the lateral malleolus and the posterior fragment. The posterior fragment is fixed with lag screws or a buttress or antiglide plate. The lateral and medial malleolus are fixed in the same manner as in the first group. After fixation the syndesmosis is tested by a bone hook or external rotatory stress under fluoroscopic control. If the syndesmosis is unstable, two transsyndesmotic positioning screws are placed. Non-weightbearing mobilization is instructed for 6 weeks after operation. After 6 weeks gradual weight-bearing mobilization is allowed and physiotherapy is started. Low-molecular weight heparin 2850 international units was administered daily as long as patients are immobilized in cast.

### End points and follow-up

The primary outcome of this study is the functional outcome after 1 year assessed by the AAOS (American Association of Orthopaedic Surgeons) Foot and Ankle-score. Secondary outcome parameters include functional outcome measured with the AOFAS-questionnaire (American Orthopaedic Foot and Ankle Society) and with the Olerud and Molander functional score and osteoarthritis on plain radiographs 1 and 5 years postoperatively. Pain measured on a Visual Analogue Scale (VAS-pain), range of motion in the upper ankle joint (dorsal and plantarflexion) measured using a goniometer and general health measured using the Euroqol-5D are assessed at each visit. The total study period for a participant is 5 years. The entire study will be performed in approximately 10 years. An overview of all measurements during the follow-up period is provided in Table 2. Patients visit the outpatient clinic at 6 weeks, 12 weeks, 26 weeks, 1 year and 5 years after surgery. Deviations of the treatment protocol will be reported.

**Table 2:** Measurements during follow-up

	Post-operative	6 weeks	12 weeks	26 weeks	1 year	5 years
*X-ray*	×	×			×	×
*CT-scan*	×					
*Olerud & Molander*		×	×	×	×	
*AAOS*				×	×	×
*AOFAS*				×	×	×
*Euroqol-5D*		×	×	×	×	×
*VAS-pain*		×	×	×	×	×
*Range of Motion*			×	×	×	X

### Sample size

In this study functional outcome as measured by the AAOS score will be used as the primary outcome parameter. Up to now the minimal clinically important difference has not been determined and published for the AAOS score. Recently, a Cochrane Review suggested a difference of 10 points to be clinically relevant [24]. For the sample size calculation we adopted this 10 point difference, with a standard deviation of 15 points and a significance level of 5%. To achieve 80% power, group samples of at least 36 patients are needed. To account for 15% drop-out, group samples of 42 patients are needed (84 in total).

### Statistical analysis

The analysis will be performed on the basis of the intention-to-treat principle. Baseline characteristics (age, gender, fragment size, dislocation posterior fragment etc.) of the study groups will be described using summary statistics. Continuous outcome measures (Olerud and Molander ankle score, VAS-pain, AAOS, range of motion) will be reported as mean and standard deviation and will be compared between the treatment groups by an unpaired t-test. Multiple imputation for missing data will be performed. Linear mixed models will be used to compare the functional outcome of the two groups during the follow-up.

Data will be analyzed using the “Statistical Package for the Sciences” version 22.0 or higher. Statistical testing will be 2-tailed and a p-value <0.05 will be used as threshold for statistical significance.

## Discussion

This study seeks answers to the question whether or not open anatomical reduction and fixation of medium-sized posterior malleolar fractures via a posterolateral approach leads to better functional outcome than when the fragment is left untouched.

Since the treatment involves two different surgical procedures with different scars from which the treatment can be deduced, randomisation status will not be blinded. Also post-operative X-rays will show different implants and therefore partial blinding in the post-operative treatment phase will not be possible.

For the pre-operative measurement of posterior fragment size and therefore patient selection, we use the Picture Achieving and Communication System (PACS) which is standard in all participating hospitals. Two observers measure the fragment size in PACS on plain X-ray. However, in a discussion amongst clinicians the preferred device for posterior fracture fragment was recently debated [25, 26]. The most widely used and currently most reproducible one, is the method described above in PACS, which we use for this study.

Functional outcome is measured with three questionnaires. The Olerud and Molander ankle score is best available for short term functional outcome and therefore used to evaluate during the first post-operative year. The AAOS ankle questionnaire is best available functional outcome score on long term and therefore used to evaluate 1 and 5 years post-operatively. The AOFAS ankle questionnaire is build up from a questionnaire and a limited range of motion and therefore also used to evaluate 1 and 5 years post-operatively.

The first patient was included in January 2014. We expect to include the last patient in 2018. Latest follow-up visit to our outpatient clinic and the conclusion of the data-acquisition will therefore be in 2023.

## Conclusion

The POSTFIX-trial is the first multicenter randomized clinical trial worldwide to analyse the effects of open anatomical fixation of the posterior fragment in trimalleolar fractures. New guidelines on anatomical reduction and fixation of the posterior fragment can in future be based on the results of this trial.

## Abbreviations

AAOS: American academy of orthopaedic surgeons
AO: Arbeitsgemeinschaft für osteosynthesefragen
AOFAS: American orthopaedic foot and ankle society
VAS: Visual analogue scale
